# Malignant fibrous histiocytoma of the face: report of a case

**DOI:** 10.1186/1746-160X-3-36

**Published:** 2007-10-18

**Authors:** László Seper, Richárd Schwab, Sirichai Kiattavorncharoen, Andre Büchter, Ágnes Bánkfalvi, Ulrich Joos, József Piffkó, Birgit Kruse-Loesler

**Affiliations:** 1 Department of Cranio-Maxillofacial Surgery, University of Muenster, Waldeyerstr. 30, 48149 Muenster, Germany; 2 Cooperative Research Center, Semmelweis University, POB 131, 1367 Budapest, Hungary; 3 Department of Oral and Maxillo-Facial Surgery, Mahidol University, 6 Yothe Rd.Rajthevee, 10400 Bangkok, Thailand; 4 Group practice Engelke, Büchter, Immenkamp, Hohenzollernring 10, 48145 Muenster, Germany; 5 Gerhard-Domagk-Institute of Pathology, University of Muenster, Domagkstraße 17, 48149 Muenster, Germany; 6 Departments of Pathology and Neuropathology, University of Duisburg-Essen Medical School, Hufelandstraße 55, 45122 Essen, Germany

## Abstract

**Background:**

Soft tissue sarcomas in the head and neck region are rare and often present a difficult differential diagnosis. The aim of our presentation is to point out the complexity of the diagnosis, treatment and follow up.

**Case presentation:**

An eighty-seven year old female patient was referred to our unit with a fast growing brownish lump on the face. Four months beforehand, a benign fibrous histiocytoma (BFH) had been removed from the same location by excision biopsy with wide tumour-free resection margins. Excision biopsy of the recurrent lesion revealed a malignant fibrous histiocytoma (MFH). Radical tumour resection was completed by extended parotidectomy and neck dissection; the skin defect was covered by a regional bi-lobed flap. No adjuvant radio- or chemotherapy was administered. Full functional and cosmetic recovery was achieved; follow-up has been uneventful more than two years postoperatively.

**Discussion:**

Malignant transformation of BFH is extremely rare and if so, extended radical surgery may give a fair chance for a favourable outcome even in patients with advanced age.

## Background

Soft tissue tumours in the head and neck region sometimes display borderline pathological features regarding benign or malignant behaviour. Despite similar histological patterns the clinical outcome is often different and difficult to predict.

Malignant fibrous histiocytoma [MFH] is a primitive, often pleomorphic sarcoma consisting of partly fibroblast-like, partly histiocyte-like cells. It has been classified as a distinct histopathological entity since the nineteen-sixties [1]. Nowadays, it is one of the most common soft tissue sarcomas of late adult life, with a male predominance since all neoplasms of mesodermal origin previously regarded as sarcomas of uncertain origin are now included in the category of MFH [2]

Changing of the histological picture during progression of the disease and transformation of borderline-benign lesions to malignant phenotype has been described. Reported incidence of malignant transformation lies around 1% [3].

Benign fibrous histiocytoma [BFH] is the most common benign neoplasm in practically all localizations affecting the epidermis. The proliferative, atypical or borderline type is a cell-dense variant mostly growing faster and greater in size, with characteristically frequent local recurrences, reported incidence lying at 3–5%. Both BFH and its borderline variant are known not to give either haematogenous or loco-regional metastases to lymph-nodes [4].

The aim of our presentation is to point out the complexity of the diagnosis, treatment and follow up of patients with soft tissue tumours of borderline character.

## Case presentation

**Figure 1:**
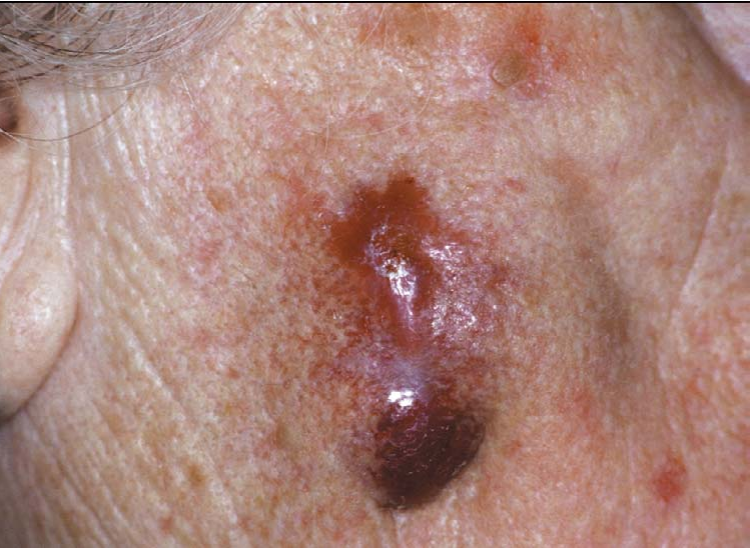
Preoperative view of the lesion on the right cheek: a smooth, firm nodule with wide erythematous border.

**Figure 2:**
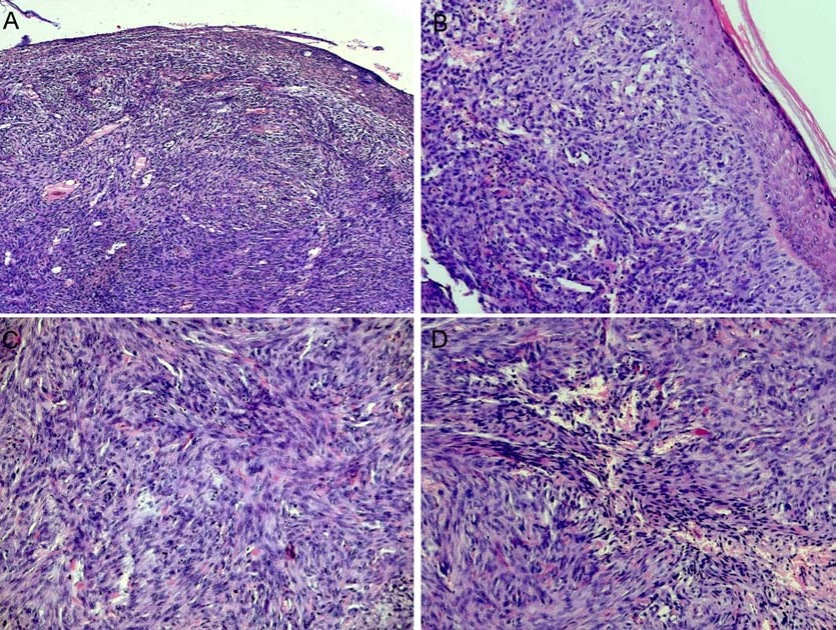
(A-D). Histopathological findings of the excision biopsy specimen from the primary benign fibrous histiocytoma: (A) The overlying epidermis is ulcerated and mildly acanthotic (H&E, 40×). (B) Subcutaneous cellular aggregation of spindle-shaped, fibroblast-like and histiocytic cells poorly demarcated from the surrounding tissues (H&E, 100×) (C) The spindled cells are arranged in interlacing fascicles forming a storiform pattern (H&E, 200×). (D) They are accompanied by varying numbers of inflammatory cells, foam cells and siderophages as well as proliferating capillaries (H&E, 200×)

An eighty-seven year old woman was referred to our department because of a fast-growing, dark-brownish, indurated skin nodule sized 3 cm in diameter, which infiltrated the skin and the deeper soft tissue layers of the face (Fig 1.) Four months beforehand, a benign cutaneous histiocytoma (BFH) had been removed from the same location by excision biopsy (Fig. 2a–d), which had been a painless, slow-growing, brownish lump not exceeding 1 cm in diameter clinically. Surgical margins had been disease-free.

**Figure 3:**
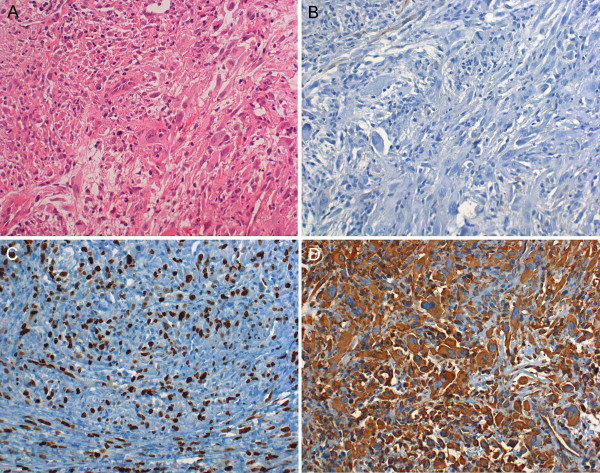
(A-D). Histopathological and immunohistochemical findings of the recurrent lesion diagnosed as malignant fibrous histiocytoma of myxoid type: (A) Heterogeneous fibroblastic spindle cells, histiocytic, inflammatory and pleomorphic giant cells are scattered throughout the tumour mass embedded in myxoid stroma containing plentiful collagen fibres. (H&E, 200×). (B) Negative immunohistochemical reaction for cytokeratins [pan-anti-cytokeratin antibody; KL-1 (Ventana, Germany)], (C) Strong positive reaction with the MIB1 antibody showing a high proliferative activity of the tumor, MIB-1 labelling index: 50%(100×), (D) Strongly positive immunohistochemical reaction for vimentin in the vast majority of tumour cells (400×)

Excision biopsy of the current lesion revealed an infiltrative, well-vascularised mesenchymal neoplasm containing pleomorphic tumour cells, increased mitotic count with some atypical mitoses, and myxoid stroma rich in collagen fibres. Immunohistochemistry was strongly positive for vimentin, and negative for cytokeratin, smooth-muscle actin, desmin, S-100 and melanoma-specific antigen. The MIB1 positive proliferative fraction was 50% (Fig. 3a–d). The histological diagnosis of a "myxoid-type malignant fibrous histiocytoma (MFH)" with tumour-free surgical margins up to 3 cm was made [pT1a, pN0 (26/0), pMx, R0]. Histological re-evaluation of the primary tumour by an independent pathologist confirmed the original diagnosis of benign fibrous histiocytoma with ulceration, inflammation and increased cellularity ("irritated BFH").

**Figure 4:**
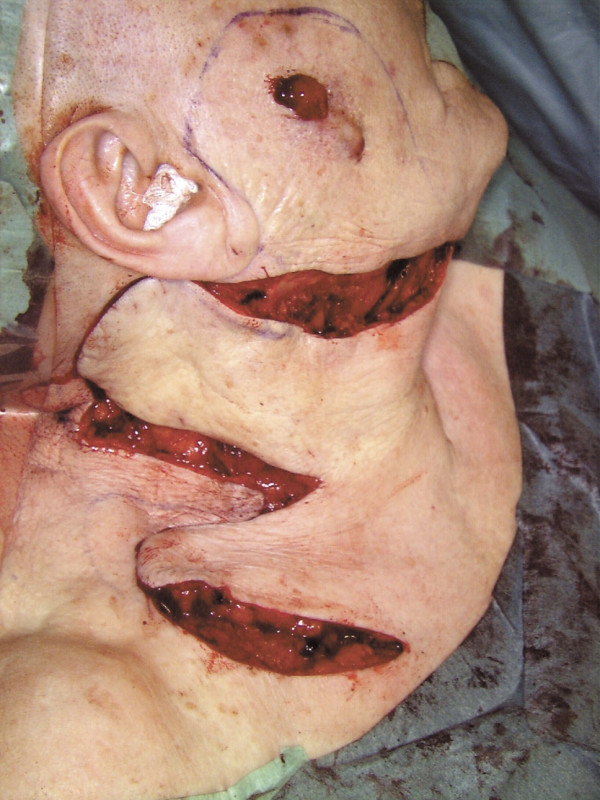
Intraoperative view after conservative neck dissection and prior to tumour removal, parotidectomy and placing of the local bi-lobed flap. The safety margin is well marked around the tumour and the preparation of the flap is completed.

**Figure 5:**
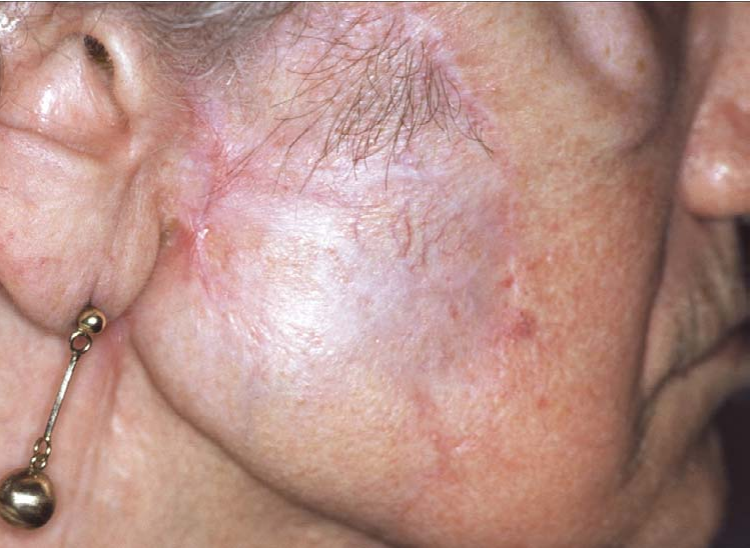
Six months postoperatively with full cosmetic and functional recovery.

Clinical staging investigations of the current malignancy [chest X-ray, MRI-scan of the neck and skull, abdominal and neck ultrasound, bone scintigraphy] showed superficial infiltration of the cutis and subcutis. Neither infiltration of the Masseter nor metastases in the regional lymph nodes or elsewhere were found. Radical excision of the tumour was completed by parotidectomy and modified neck dissection, which resulted in a cosmetic defect of the right face that was immediately corrected by a local bi-lobed flap (Fig. 4). No postoperative radio- or chemotherapy was applied. Two years postoperatively, the patient was alive and well with full cosmetic and functional recovery. (Fig. 5) She died after a heart attack three years after surgery without any signs of tumour recurrence or metastasis. Autopsy was not carried out.

## Discussion

Benign fibrous histiocytomas (BFH) are frequently found in sun-exposed skin of the extremities and of the head and neck. They are among the most common soft tissue lesions of the skin [1]. Their biological nature, in particular whether they are neoplastic or reactive, has long been disputed. Recent studies have found cytogenetic evidence of clonal chromosomal abnormalities in a part of lesions, which support their neoplastic origin [5]. In addition to the common histological pattern, a number of variants are recognized, some of which can be confused with sarcoma. Of the cellular and atypical types, about 20% are localised in the head and neck region, they usually grow faster and are greater in size than the other subtypes, and tend to recur locally (up to 26%) [4]. Reported incidence of malignant transformation is around 1% [3].

In contrast, malignant fibrous histiocytoma (MFH) is a sarcoma with both fibroblastic and histiocytic features. It has been classified as a distinct histopathological entity since the nineteen-sixties [1]. Nowadays, it is one of the most commonly diagnosed sarcomas of late adulthood, since all neoplasms previously regarded as sarcomas of uncertain origin are now included in this category [2]. MFH typically arises in the soft tissues of the extremities and retroperitoneum, the head and neck region is seldom involved. In superficial sites such as the skin, MFH may behave in a benign fashion despite high-grade looking and fast growing tumour cells.

The primary treatment of MFH consists of radical excision with wide safety margins and dissection of the loco-regional lymph nodes [6]. Post- or sometimes even preoperative radio- or chemotherapy may be an adjuvant option of treatment, however their indications and efficacy remain controversial [2,6]. In the present case, we have disregarded adjuvant oncological treatment because of the age of the patient and the lack of clear-cut evidence for indication.

In conclusion, predicting biological behaviour on the base of cellular features in cutaneous fibrohistiocytic tumours is sometimes very difficult. It is well illustrated by the present case, which showed malignant transformation in recurrence despite the bland histopathological appearance of the primary lesion. It is therefore mandatory that patients with such lesions are closely monitored postoperatively, so as to be able to act promptly in case of local recurrences. Patients' management requires close collaboration between dermatologists, maxillo-facial surgeons, radiologists and pathologists, which may result in sufficient clinical outcome.

## Authors' contributions

LS and RS analysed the case, reviewed all patient data and drafted the manuscript. All authors were involved in planning the treatment. The excision biopsy, the tumour removal and the neck dissection was carried out by SK, JP whereas BKL carried out the plastic reconstruction of the face. AB and ABü did the histological and immunhistological analysis and contributed to the final conclusions of the case report. UJ suggested the idea for the case report and reviewed and contributed to the writing of final version. JP, AB, ABü and BKL contributed substantially to discussions and in writing of the paper. All authors reviewed the paper for content, and contributed to the writing of all iterations of the paper, including the final version of the manuscript. All authors approved the final report.
